# Plant trait networks reveal adaptation strategies in the drylands of China

**DOI:** 10.1186/s12870-023-04273-0

**Published:** 2023-05-19

**Authors:** Xiaoting Wang, Mingfei Ji, Yahui Zhang, Liang Zhang, Muhammad Adnan Akram, Longwei Dong, Weigang Hu, Junlan Xiong, Ying Sun, Hailin Li, Abraham Allan Degen, Jinzhi Ran, Jianming Deng

**Affiliations:** 1 State Key Laboratory of Herbage Improvement and Grassland Agro-ecosystems, College of Ecology, Lanzhou University, Lanzhou, 730000 China; 2 Collaborative Innovation Center of Water Security for Water Source Region of Mid-Route Project of South-North Water Diversion of Henan Province, College of Water Resource and Environment Engineering, Nanyang Normal University, Nanyang, 473061 China; 3 School of Economics, Lanzhou University, Lanzhou, 730000 China; 4 Desert Animal Adaptations and Husbandry, Wyler Department of Dryland Agriculture, Blaustein Institutes for Desert Research, Ben-Gurion University of Negev, Beer Sheva, 8410500 Israel

**Keywords:** Plant functional traits, Interdependence, Plant traits networks, Adaptation strategies, Arid environment

## Abstract

**Background:**

Plants accomplish multiple functions by the interrelationships between functional traits. Clarifying the complex relationships between plant traits would enable us to better understand how plants employ different strategies to adapt to the environment. Although increasing attention is being paid to plant traits, few studies focused on the adaptation to aridity through the relationship among multiple traits. We established plant trait networks (PTNs) to explore the interdependence of sixteen plant traits across drylands.

**Results:**

Our results revealed significant differences in PTNs among different plant life-forms and different levels of aridity. Trait relationships for woody plants were weaker, but were more modularized than for herbs. Woody plants were more connected in economic traits, whereas herbs were more connected in structural traits to reduce damage caused by drought. Furthermore, the correlations between traits were tighter with higher edge density in semi-arid than in arid regions, suggesting that resource sharing and trait coordination are more advantageous under low drought conditions. Importantly, our results demonstrated that stem phosphorus concentration (SPC) was a hub trait correlated with other traits across drylands.

**Conclusions:**

The results demonstrate that plants exhibited adaptations to the arid environment by adjusting trait modules through alternative strategies. PTNs provide a new insight into understanding the adaptation strategies of plants to drought stress based on the interdependence among plant functional traits.

**Supplementary Information:**

The online version contains supplementary material available at 10.1186/s12870-023-04273-0.

## Introduction

Plant functional traits are measurable attributes that are closely related to plant functions [1]. Plant traits are not independent of each other, as there is a close relationship between them [2, 3]. In addition, the trait-environment relationships and trait covariation can also be quantified [4–6]. For example, the leaf economics spectrum (LES) is a general concept describing the coordinated variation of leaf structural, chemical and physiological traits across a resource gradient [7, 8]. However, most previous reports focused on the coordination across plant traits, but ignored the complex relationships between multiple traits. Combination of plant traits contribute to multi-functional systems [9], enabling plants to alter strategies during development in order to cope with environmental changes and resource competition [2, 10, 11]. Therefore, visualizing the complex network relationships between multiple plant traits can enhance our understanding of how plants adapt to their environment.

Previous studies revealed the interdependence of multiple traits by employing correlation analyses [12, 13], structural equation models [14–16] and principal component analyses [4, 6, 17, 18]. These quantitative methods are limited in assessing the interdependence among multiple traits in plants [19]. However, network analysis is an effective method to quantify complex relationships of multiple traits. Network analysis was used to visualize the interdependence between multiple traits and ecological network parameters, such as degree, edge density and modularity, to describe the adaptation strategies of plants [9, 19–21]. A high degree of a trait (i.e., a hub trait) is the selection of a trait due to the environment that changes the plant’s phenotype to a large extent [21]. The network with a high edge density indicates a close relationship between traits and the synergism of multiple traits, which means that plants can perform their functions more efficiently [9, 19]. The network with high modularity refers to the differentiation of plant traits into different functional modules, and each module performs different functions [21, 22].

Recent studies applied network analysis to identify both the relationships among plant traits and the hub or key traits of plants under different environmental conditions [9, 19, 20]. For example, by employing parameters network analysis, Li et al. concluded that leaf lifespan and leaf nitrogen content are hub traits in the global economic trait dataset based on leaf trait networks [19]. Kleyer et al., using a network of trait correlations, identified biomass allocation traits and stem specific length as key traits in herbs [20]. Flores-Moreno et al. reported that terrestrial plants had a strong correlation among traits, and that leaf lifespan and stem specific density displayed high centrality in the network [21].

Drylands, which are highly vulnerable to climate change, account for approximately 45% of the global land area [23]. They provide important ecosystem functions and services including carbon, nitrogen and water cycling [24–26]. Crucial ecological functions, such as resource acquisition and conservation, are tightly linked to plant functional traits [2]. Therefore, it is important to improve our understanding of multiple plant traits relationships in drylands. In dryland ecosystems, plants are expected to favor conservative traits, such as slower photosynthetic rate, higher leaf mass per area (LMA) and longer lifespan [27]. To resist water and nutrient stresses in drylands, perennial plants increase root proliferation and length to enhance water uptake at the expense of reducing allotment of nutrients to the above-ground part of the plant [28–32]. The adaptation of plants to drought and low nutrients has led to a coordinated diversity among different organs in the utilization and acquisition of nutrients [4, 33]. In addition, plants generally adapt to the environment by modifying their functional traits of leaves [34]. Consequently, an analysis of multiple traits in different plant organs could determine adaptation strategies in dryland ecosystems.

The adaptation strategies of plants to drought also depend on their life-forms [35]. Resource acquisition and adaptations to the environment in woody and herbaceous plants have been well studied in drylands of China [31, 32]. For example, herbaceous plants, in particular annual herbs with fast growth rates and short lifespans, often require more resources and nutrients and display a lower tolerance to adverse conditions, such as aridity, soil alkalinity, and soil nutrient deficiency, than woody plants [36, 37]. Thus, herbaceous plants serve as negative indicators, and woody plants serve as positive indicators of increasing aridity [32]. However, the interdependence of multiple traits in herbaceous and woody plants in the drylands of China remains unclear. Moreover, Berdugo et al. demonstrated that important thresholds of ecosystem functional traits emerge along a drought gradient [38]. Recently, we reported that a similar shift in response to drought stress exists in the drylands of China at an aridity level of approximately 0.8 (1-AI, where AI is the aridity index), in plant and microbial diversity, plant and soil functional traits, and biodiversity-soil multi-functionality relationships [32, 39–41]. Moreover, herbaceous species are dominant in low arid regions (AI > 0.2) and woody species are dominant in high arid regions (AI < 0.2), and their adaptation strategies are distinct to drought stress [31, 40, 41]. Although the functional traits of plants in drylands have been widely reported [28, 29, 32, 34, 42], plant trait networks remain unclear. The aim of this study was to fill this knowledge gap.

Sixteen plant functional traits, of which six economic traits, six chemical traits and four structural traits, were measured in 80 dominant plant species from 83 sites in the dryland ecosystems of China. Network analysis was used to determine: (1) the network relationships of functional traits and their adaptation strategies to drought stress across different arid regions and plant life-forms; (2) the key traits among the sixteen leaf traits; and (3) the connectivity of economic, chemical and structural traits across different arid regions and plant life-forms.

## Materials and methods

### Study area, sampling and measurements

This study included 83 field sites across drylands of China (Figure S1). The sites were typical temperate drylands, with an aridity index (AI) ranging between 0.02 and 0.51. The natural vegetation types included desert shrubs, desert steppe and temperate steppe [40, 41].

Field investigations and samplings were conducted during the growing seasons (June to September) from 2013 to 2017, using standardized protocols described by Chen et al. and Deng et al. [28, 31]. At least five individual plants of each species were collected in each of three random quadrats (each 30 m × 30 m) at each sampling site [32, 39, 41]. Sixteen plant traits were measured and classified into three types based on function, namely, economic, chemical and structural. Economic traits included area-based photosynthetic rate (A_area_), leaf mass per area (LMA), leaf thickness (LT), leaf nitrogen concentration (LNC), leaf carbon concentration (LCC) and leaf phosphorus concentration (LPC) [27, 43]. Chemical traits included root carbon concentration (RCC), root nitrogen concentration (RNC), root phosphorus concentration (RPC), stem carbon concentration (SCC), stem nitrogen concentration (SNC) and stem phosphorus concentration (SPC) [32, 44]. Structural traits included leaf tissue density (LD), leaf volume (LV), leaf dry matter content (LDMC) and leaf area (LA) [8]. The measurements and classification of plant traits are presented in Appendix S1, and the abbreviations and units of these traits are listed in Table S1.

### Establishment of plant trait networks

Plant trait networks (PTNs) are biological networks that use plant traits as nodes and relationships between the traits as edges [19]. Firstly, Pearson correlations were calculated between traits. Secondly, a threshold of pairwise correlations was set, where *P* < 0.05 was retained and set to 1, and other relationships were set to zero [19]. The adjacency matrix A = [a_i, j_] was obtained with ${\text{a}}_{\text{i,j}}\in\text{[0,1]}$. If the relation value of a pairwise trait-trait relationship was 1, then the two traits were connected by edge; however, if the relationship was 0, then the two traits were not connected by edge.

### Parameters of plant trait networks

Three network parameters, that is, degree, edge density and modularity, were considered due to their ecological importance [21]. The degree is the sum of edges that connect focal node traits to other nodes. A trait with a high degree is considered a hub trait [19], which is beneficial to resource acquisition and effective utilization within and across plant tissues [19, 45]. Edge density is the ratio of the sum of the actual edges to the sum of the largest possible edges, and ranges from zero to one. Plant trait networks with a high edge density represent an efficient access and mobilization of resources (as all traits are closely connected) [21]. Modularity describes the degree of separation between sub-networks (or modules) [46]. Plant trait networks with higher modularity values have tighter internal connections of the module and looser external connections [21], which confer an advantage under variable conditions, as it provides robustness [22, 47].

### Statistical analyses

The parameters of PTNs were calculated using the “igraph” package in R. A PTN was established for each bootstrapping by performing 5000 random resamplings to determine the range of uncertainty for these network parameters, and at least three-fourths of all species were selected randomly for each time. In addition, previous studies have shown that the number of species affects the network relationships between traits [43]. Therefore, to test the dependence of plant trait networks on the number of species, we simulated the entire dataset combining species from all communities. A replacement sampling method selected from 10 to 188 random species determined 500 PTNs for each combination and calculated their PTN level parameters. The means of the PTN level parameters were plotted against the number of species.

To compare the importance of economic (A_area_, LMA, LT, LCC, LNC and LPC), chemical (RCC, RNC, RPC, SCC, SNC and SPC) and structural (LA, LD, LV, and LDMC) traits, the absolute and relative importance were calculated. The absolute importance was calculated as the average degree of each type of trait, and the relative importance was calculated as the absolute importance divided by the sum of all trait degrees [48]. A Duncan’s multiple range test was used to compare network parameter means among plant traits. An independent sample t-test compared plant trait networks between different life-forms (woody and herbaceous plants) and aridity regions (sites with aridity index < 0.2 and > 0.2, that is, arid regions and semi-arid regions).

Trait data were log-transformed before analysis, and all statistical analyses and visualizations used R software (version 4.0.3, 2020). A level of *P* < 0.05 was accepted as significant.

## Results

### Plant trait networks in the drylands of China

**Fig. 1:**
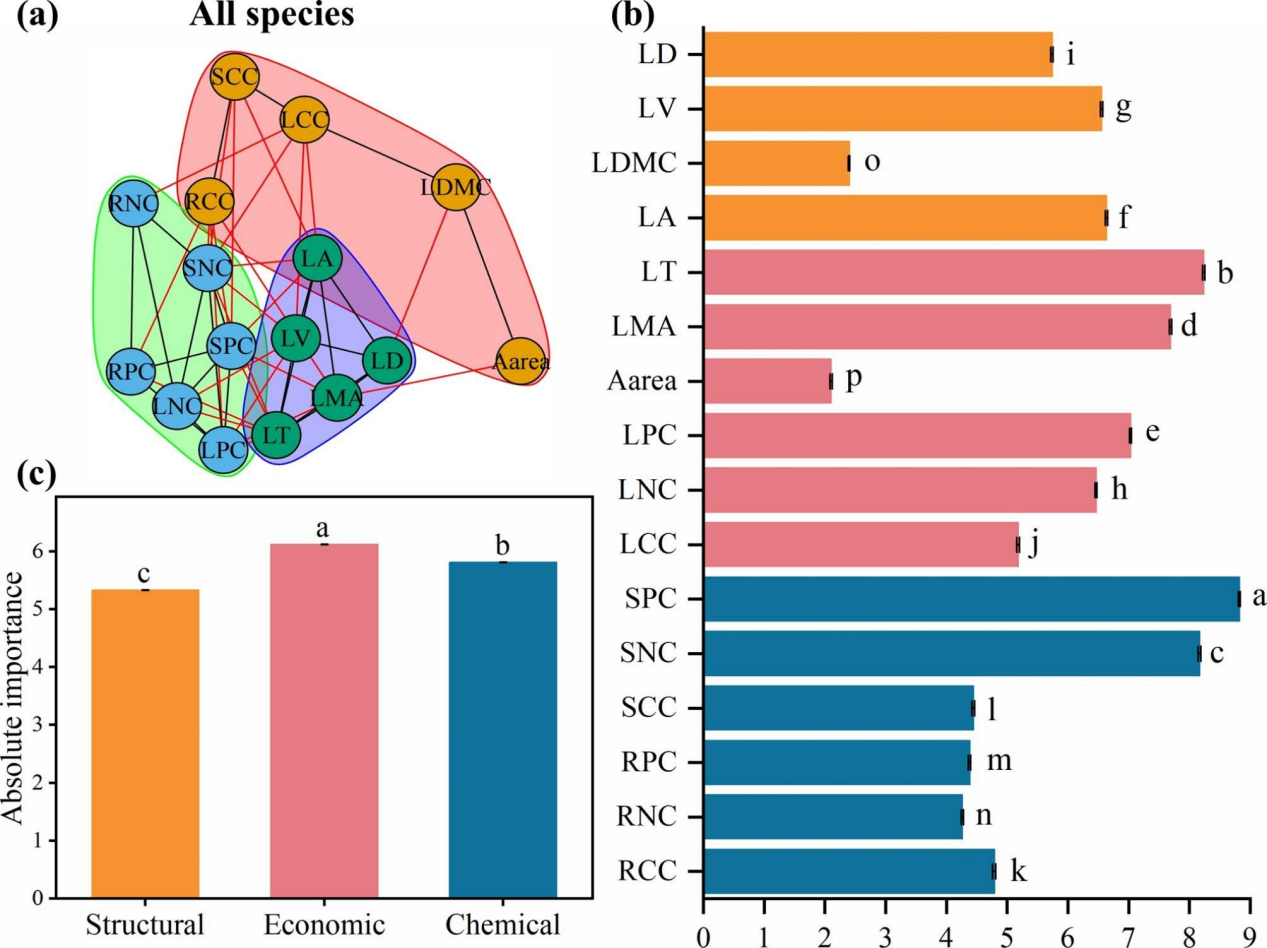
Trait networks of 16 plant traits including leaf tissue density (LD), leaf volume (LV), leaf dry matter content (LDMC), leaf area (LA), leaf thickness (LT), leaf mass per area (LMA), area-based photosynthetic rate (A_area_), leaf phosphorus concentration (LPC), leaf nitrogen concentration (LNC), leaf carbon concentration (LCC), stem phosphorus concentration (SPC), stem nitrogen concentration (SNC), stem carbon concentration (SCC), root phosphorus concentration (RPC), root nitrogen concentration (RNC), and root carbon concentration (RCC) for all species in the drylands of China. (**a**) Modularity; (**b**) Degree; and (**c**) Absolute importance of structural, economic and chemical traits. Traits with the same background color belong to the same module. The black and red edges represent connections within and between modules, respectively. Structural, economic and chemical traits are represented by orange, red, and blue colors, respectively. Means with different letters differ from each other (*P* < 0.05). Error bars represent standard error (SE)

Based on the dataset of the sixteen plant traits, plant trait networks were constructed and parameters were calculated (Fig. 1a). Edge density averaged 0.34 and ranged between 0.24 and 0.48, while modularity averaged 0.22 and ranged between 0.11 and 0.37 (Tables 1 and 2). Degree differed significantly for all sixteen traits (*P* < 0.05 for all, Fig. 1b). SPC was the most important factor in the plant trait networks (Fig. 1b, Table S2), and economic traits were more important than either chemical or structural traits (Fig. 1c, Table S3). The PTN parameters were highly sensitive to species numbers (Figure S2a-d), and the edge density and modularity of the PTNs increased and decreased, respectively, with increased species numbers.

**Table 1:** Variation in the modularity of plant trait networks (PTNs) for all species, different plant life-forms and arid regions

Group	Mean	Standard deviation	Standard error	Minimum	Maximum
**PTNs-all species**	0.22	0.033	0.0005	0.11	0.37
**PTNs-woody plants**	0.30	0.039	0.0006	0.17	0.45
**PTNs-herbaceous plants**	0.22	0.028	0.0004	0.12	0.33
**PTNs-arid regions**	0.24	0.029	0.0004	0.14	0.33
**PTNs-semi-arid regions**	0.22	0.035	0.0005	0.08	0.33

**Table 2:** Variation in edge density of plant trait networks (PTNs) for all species, different plant life-forms and arid regions

Group	Mean	Standard deviation	Standard error	Minimum	Maximum
**PTNs-all species**	0.34	0.032	0.0005	0.24	0.48
**PTNs-woody plants**	0.29	0.030	0.0004	0.20	0.40
**PTNs-herbaceous plants**	0.38	0.028	0.0004	0.26	0.48
**PTNs-arid regions**	0.28	0.023	0.0003	0.21	0.38
**PTNs-semi-arid regions**	0.45	0.042	0.0006	0.35	0.63

### Woody and herbaceous plant trait networks in the drylands of China

**Fig. 2:**
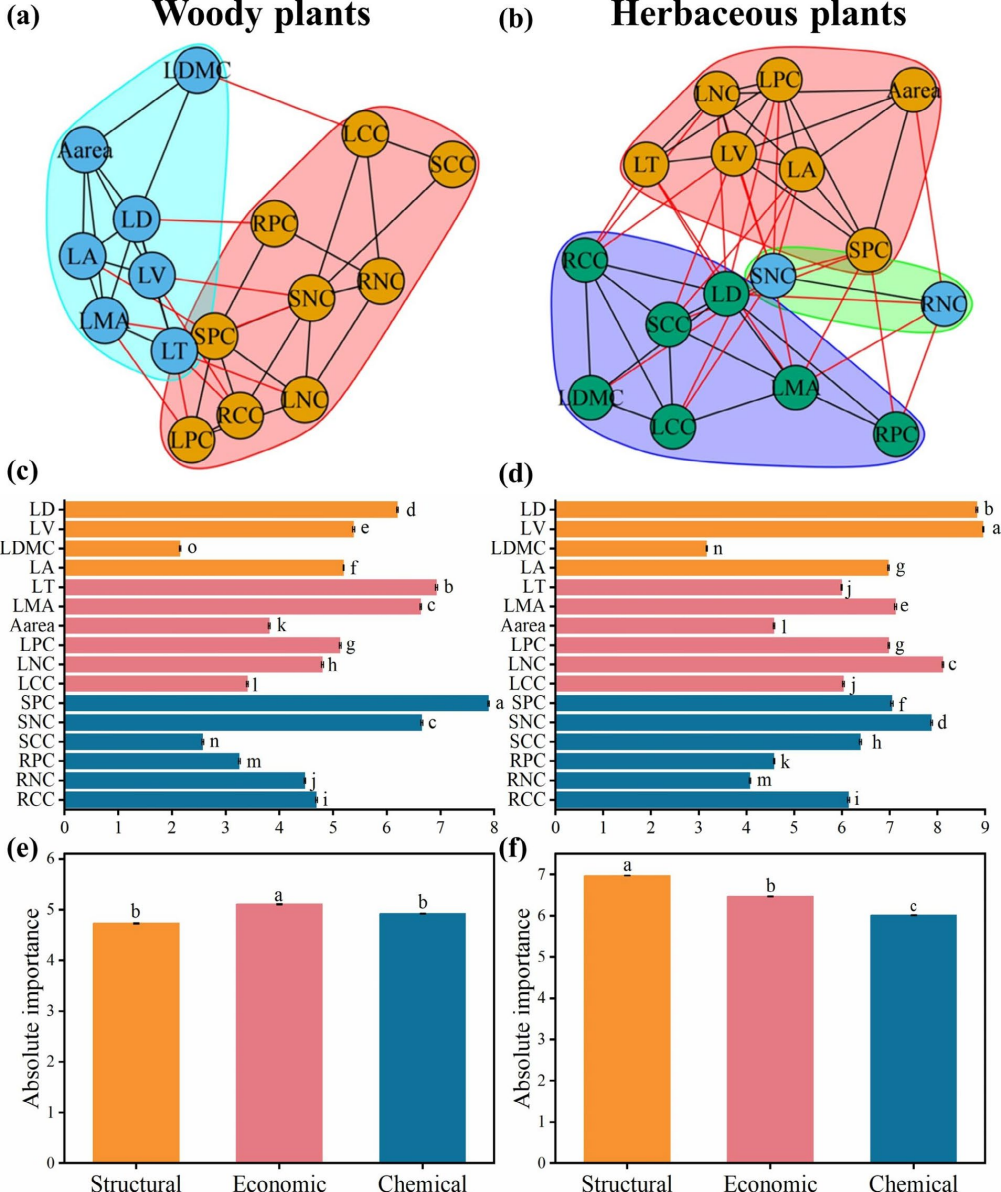
Trait networks of 16 plant traits including leaf tissue density (LD), leaf volume (LV), leaf dry matter content (LDMC), leaf area (LA), leaf thickness (LT), leaf mass per area (LMA), area-based photosynthetic rate (A_area_), leaf phosphorus concentration (LPC), leaf nitrogen concentration (LNC), leaf carbon concentration (LCC), stem phosphorus concentration (SPC), stem nitrogen concentration (SNC), stem carbon concentration (SCC), root phosphorus concentration (RPC), root nitrogen concentration (RNC), and root carbon concentration (RCC) for woody and herbaceous plant life-forms in the drylands of China. Modularity for woody plants (**a**) and herbaceous plants (**b**), and degree for woody plants (**c**) and herbaceous plants (**d**). Traits with the same background color belong to the same module. The black and red edges represent connections within and between modules, respectively. Absolute importance of structural, economic and chemical traits for woody plants (**e**) and herbaceous plants (**f**). Structural, economic and chemical traits are represented by orange, red, and blue colors, respectively. Means with different letters differ from each other (*P* < 0.05). Error bars represent standard error (SE)

Differences within the plant trait networks were determined for the different plant life-forms (Fig. 2a-b). The edge density of plant trait networks was lesser (*P* < 0.05) for woody plants (PTNs-woody plants) than for herbaceous plants (PTNs-herbaceous plants), while the modularity of woody plants was greater (*P* < 0.05) than for herbaceous plants (Tables 1 and 2; Fig. 2a-b, Figure S3a-b). SPC had the highest degree of connection with other traits for PTNs-woody plants, while LV had the highest degree of connection with other traits for PTNs-herbaceous plants (Fig. 2c-d, Table S4). Economic traits were most important for woody plants, whereas structural traits were most important for herbaceous plants (Fig. 2e-f, Table S3).

### Differences in plant trait networks among different arid regions

**Fig. 3:**
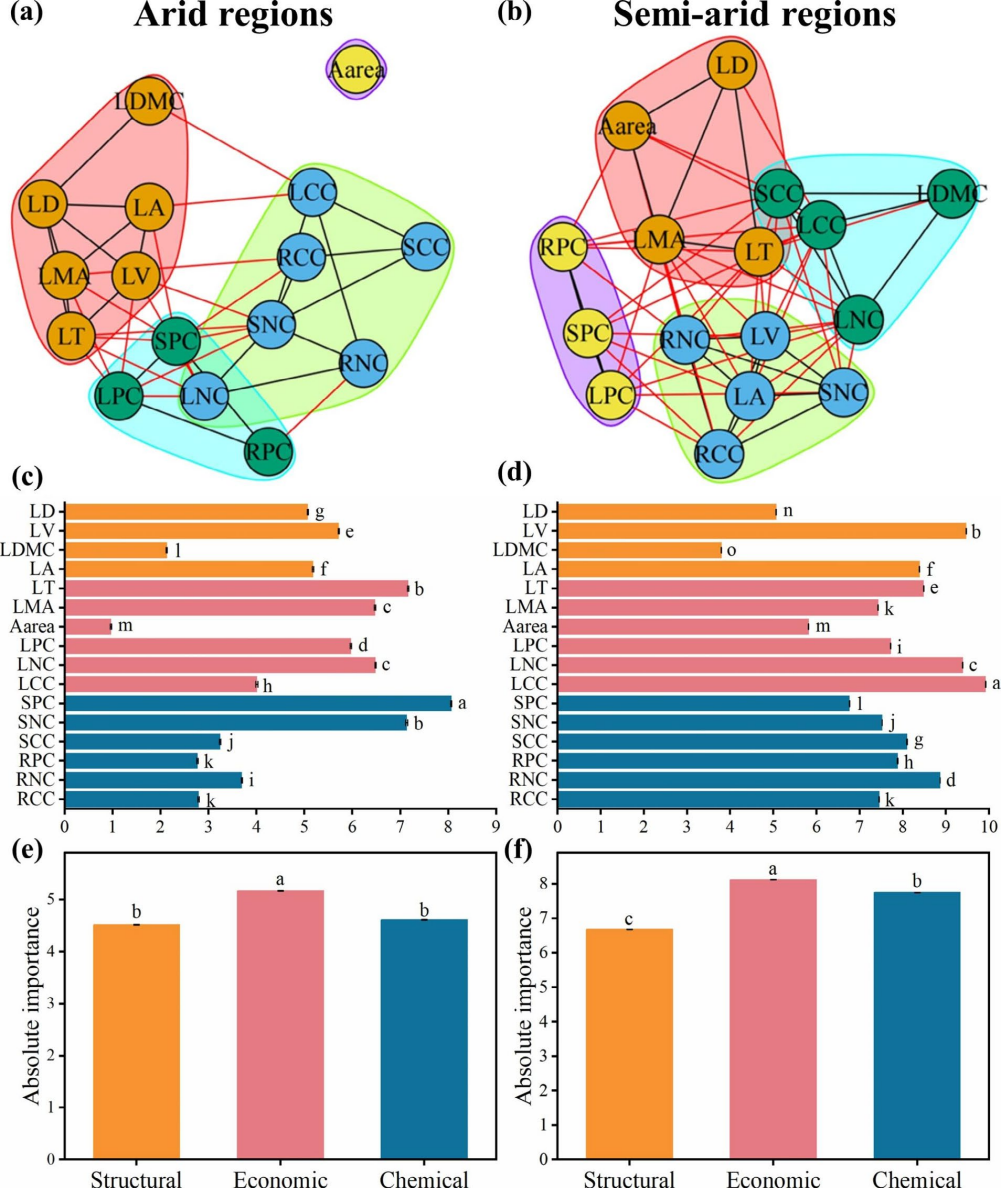
Trait networks of 16 plant traits including leaf tissue density (LD), leaf volume (LV), leaf dry matter content (LDMC), leaf area (LA), leaf thickness (LT), leaf mass per area (LMA), area-based photosynthetic rate (A_area_), leaf phosphorus concentration (LPC), leaf nitrogen concentration (LNC), leaf carbon concentration (LCC), stem phosphorus concentration (SPC), stem nitrogen concentration (SNC), stem carbon concentration (SCC), root phosphorus concentration (RPC), root nitrogen concentration (RNC), and root carbon concentration (RCC) for arid and semi-arid regions in drylands of China. Modularity for arid regions (**a**) and semi-arid regions (**b**), and degree for arid regions (**c**) and semi-arid regions (**d**). Traits with the same background color belong to the same module. The black and red edges represent connections within and between modules, respectively. Absolute importance of structural, economic and chemical traits for more arid regions (**e**) and less arid regions (**f**). Structural, economic and chemical traits are represented by orange, red, and blue colors, respectively. Means with different letters differ from each other (*P* < 0.05). Error bars represent standard error (SE)

Differences in PTNs were examined in different arid regions (Fig. 3a-b). Edge density of plant trait networks was lesser in arid regions (i.e. AI < 0.2) (PTNs-arid regions) than semi-arid regions (i.e. AI > 0.2) (PTNs-semi-arid regions) (Table 2). The composition and number of modules in different arid regions were inconsistent (Fig. 3a-b), while the degree of modularity in arid and semi-arid regions were similar (Table 1). SPC and LCC had the highest degree of connection with other traits for PTNs-arid regions and PTNs-semi-arid regions, respectively (Fig. 3c-d, Table S5). Compared with the chemical or structural traits, the absolute and relative importance of the economic traits were greater for all plants in both arid and semi-arid regions (Fig. 3e-f, Table S3).

## Discussion

### Connections among traits across drylands of China

Terrestrial plants have a high connectedness among plant traits on a global scale [21]. In contrast, the present study revealed a relatively low proportion of connections among the 16 plant traits in the drylands of China (Table 2). Edge density are connected to trade-offs between connection costs and efficiency [22, 49]. In a harsh environment, the variation range of traits is narrow, which results in a weak relationship between traits [21]. Therefore, adverse conditions, such as drought, soil nutrient depletion and high temperature reduce trait connectivity in the drylands of China. Substantial differences between herbaceous and woody plants were also observed in the proportion of connections among traits. This can result from physiological and anatomical trait differences between these life-forms [21], and species-richness differences in the local environment [40].

In addition, the present study demonstrated a strong positive relationship between species richness and edge density (Figure S2a-b), and that the edge density was greater in semi-arid than in arid regions (Table 1). Trait network correlations can reflect interactions of plant traits and even plant species, which are, therefore, linked with species richness [43, 50]. In the dryland ecosystems in China, plant diversity decreases with increasing aridity, with a concomitant shift in dominant species from herbs to shrubs [40, 41]. The change of dominant species and the decrease in species diversity may lead to a lower edge density in arid than in semi-arid regions.

### Differences in plant trait networks across drylands of China

Modules are groups of traits that are closely related and perform a specific function [22]. Here, at least two modules were present in the plant trait networks in the drylands of China (Figs. 1a, 2a-b and 3a-b), which is consistent with the studies of Ackerly and Díaz et al., who identified at least two independent axes of trait variation when describing the plant trait strategy dimension [2, 51]. However, it is important to note that some plant trait combinations, such as LNC and LPC, were not always in the same module (Figs. 1a, 2a-b and 3a-b). This suggests that trait combinations in modules are not always representative [20], and different trait combinations modes can produce equivalent fitness values to adapt to the environment [52]. Therefore, plants can adapt to environments by alternative strategies, that is, by different combinations of traits [21, 48, 53]. Moreover, modularity is correlated strongly with the number of species (Figure S2c-d). This supports the theoretical expectation that network complexity increases with species richness [43], and suggests that PTNs are simpler (e.g. high modularity) in systems with limited species richness.

In the current study, modularity was greater in woody than in herbaceous plants (Table 1), suggesting that the modules in the plant trait networks in woody plants are more independent from one another than in herbaceous plants. Plant multiple traits with high modularity can provide plants with more flexibility to adjust functions to changing environments [21, 54], and, consequently, woody plants are better adapted to drought conditions than herbaceous plants. In contrast to woody plants, herbaceous plants have a short lifespan, are highly sensitive to climate change [55], and display a high species richness across drylands [40], which, in combination, may result in lower modularization and less independence from one another compared to woody plants. Furthermore, module compositions differed among arid regions, but the degree of modularity in arid and semi-arid regions were similar (Table 1), indicating that the level of drought has little influence on modularity. In conclusion, module compositions and the degree of modularity vary with species richness and plant life-forms, enabling plants to use different strategies to cope with the environment.

### Highly connected traits in the plant trait networks in drylands of China

A highly connected trait (i.e. a hub trait) means that the trait is highly related to other traits in a network, which suggests the trait may regulate pivotal functions affecting the entire phenotype [56]. Stem phosphorus concentration (SPC) was the trait with the most connections to other traits across the drylands of China (Fig. 1b, Table S2). Phosphorus in leaves is essential for plant growth and metabolism, especially for photosynthetic carbon assimilation [57], and stems provide storage for P, which means that stems are especially critical for plant respiration and nutrient cycling [58]. Previous studies reported a high level of soil total phosphorus (STP), but a low level of soil available phosphorus (SAP) in the temperate deserts of north-west China [59], and that the plants in the drylands of China may be limited by phosphorus [32]. Phosphorus in woody stems was most sensitive to variation in soil nutrient availability, and plants can use nutrients stored in stems to fulfill leaf needs when nutrients are limited [60]. This could explain why stem phosphorus content is a hub trait in the drylands of China.

Trait centrality was altered within the plant’s life-forms and in different arid regions. Leaf carbon concentration (LCC), an important trait reflecting leaf structural energy costs [61, 62], was the key trait for plant growth in semi-arid regions, and leaf volume (LV) was the key trait for herbaceous plants in drylands of China (Figs. 2d and 3d). High structural investments, that is high leaf C and lignin concentrations and a large cell wall fraction, can result in a high biomass cost of leaf construction per unit area, high resistance to herbivore attacks and long leaf lifespan [30, 63, 64]. This suggests that high-cost leaves with high C and LV is preferred in semi-arid regions. Changes in hub traits of the biological network across plant life-forms and environmental conditions may indicate the scale-dependent nature of traits [21].

### Effects of different life-forms and arid regions on the establishment of trait relations

In the present study, the sixteen plant functional traits were classified broadly as structural, chemical and economic. Economic traits were more important than the other traits in the plant trait networks across the study area (Figs. 1c and 3e-f, Table S3). Nutrient and water availability in the drylands of China are relatively scarce [65], thus, plants prioritize the connections of economic traits to improve the efficiency of storing carbon and nitrogen to resist shortages and enable the plant to be more competitive [66]. In both arid and semi-arid regions, the importance of economic traits was higher than structural and chemical traits, which suggested that plants invest more resources to economic traits in an arid environment. This would result in the connection of crucial related traits to optimize resource allocation [7].

The importance of economic, chemical and structural traits varied across different life-forms; the connectivity of economic traits was important for woody plants, whereas, the connectivity of structural traits was important for herbaceous plants (Fig. 2e-f, Table S3). These results suggest that plant life-forms contribute to the establishing of linkages [43]. Compared with herbaceous plants, the strong tolerance to arid stress led woody plants to adopt a cost-effective strategy. For example, woody plants adapt to drought conditions by increasing LMA, reducing water consumption and increasing carbon uptake [27]. Therefore, woody plants prioritize connections between economic traits to adapt to drought stress, whereas herbaceous plants prioritize connections between structural traits to improve leaf structural robustness and reduce physical damage caused by drought [8, 67].

## Conclusions

This study provided the first evidence that adaptation strategies of plants in drylands of China are mediated by plant trait networks. Herbaceous plants had a greater edge density and a lesser modularity than woody plants, thus, woody plants are more tolerant of aridity stress than herbs. Herbaceous plants prioritize connections between structural traits and woody plants prioritize connections between economic traits. Plants displayed a greater edge density in semi-arid than in arid regions, but the degree of modularity in arid and semi-arid regions were similar, indicating plants in semi-arid regions could be more efficient for multiple functions than plants in arid regions. Stem phosphorus concentration (SPC) was a hub trait as it shared high connections with all other traits. Changes in trait modules indicated that plants adapted to the local conditions through alternative strategies. In conclusion, by using the plant trait networks (PTNs), this study provided an effective trait-based approach to explore how plants respond to the arid environment.

## Electronic supplementary material

Below is the link to the electronic supplementary material.

Supplementary Material 1

## Data Availability

Datasets are available from the corresponding author on reasonable request.
