# Supplementary material for: Plant trait networks reveal adaptation strategies in the drylands of China
Source: BMC Plant Biol. 2023 May 19;23:266. doi: 10.1186/s12870-023-04273-0 (PMC10197480; doi:10.1186/s12870-023-04273-0)

**Supplementary Information**

**Article title: Plant trait networks reveal adaptation strategies in the drylands of China**

Authors: Xiaoting Wang†, Mingfei Ji†, Yahui Zhang, Liang Zhang, Muhammad Adnan Akram, Longwei Dong, Weigang Hu, Junlan Xiong, Ying Sun, Hailin Li, Abraham Allan Degen, Jinzhi Ran, Jianming Deng^*^

**Supplementary Information**

**Additional file 1: Appendix S1.** Classification and measurements of plant traits. **Appendix S2.** The list of plant species in the study. **Figure. S1** The sampling sites (black dots) in the drylands of China. **Figure. S2** Relationships of plant trait network-level parameters to the numbers of species for simulated networks. **Figure. S3** Differences in the modularity (a) and edge density (b) of plant trait networks (PTNs) between different plant life-forms. **Figure. S4** Differences in the modularity (a) and edge density (b) of plant trait networks (PTNs) between different arid regions. **Table S1.** List of plant traits and their categories, units and abbreviations. **Table S2.** Variation in the degree of plant trait networks (PTNs) for all species among different species. **Table S3.** Comparison of the relative importance of structural, economic and chemical traits for all species, different plant life-forms and arid regions. **Table S4.** Variation in the degree of plant trait networks (PTNs) for all species among different plant life-forms. **Table S5.** Variation in the degree of plant trait networks (PTNs) for all species among different arid regions.

**Appendix S1. Classification and measurements of plant traits**

Sixteen plant traits were measured and classified into three types based on function, namely, economic, chemical and structural. Economic traits were related strongly to the carbon economy of plants, and the optimal allocation of resources through trade-offs between leaf traits and survival, growth and reproduction [1, 2]. Economic traits in the study included A_area_, LT, LMA, LCC, LNC and LPC according to Wright et al. [1] and Li et al. [3]. The content of chemical elements in different organs was the basic element of plant metabolism and adaptation to the environment [4]. The C, N, and P in roots and stems were classified as chemical traits [4]. Leaf structural traits were relatively stable and related to resource acquisition and defense [5, 6]. LV, LA, LDMC, and LD were classified as structural traits [7-9].

Plant chemical traits included root carbon concentration (RCC), root nitrogen concentration (RNC), root phosphorus concentration (RPC), stem carbon concentration (SCC), stem nitrogen concentration (SNC) and stem phosphorus concentration (SPC). The air-dried leaf sample was sieved through a 0.2 mm screen, and plant C concentration was measured by the volumetric method (ferrous sulfate titration after oxidation of potassium dichromate). A 0.5 g sample leaf was placed into a dry test tube, and 10 mL of 0.136 mol/L potassium dichromate and concentrated sulphuric acid solution (K_2_Cr_2_O_7_-H_2_SO_4_) were added. The mixture was boiled, 3-4 mg of indicator (o-phenanthroline) were added, and, after cooling, was titrated with 0.2 mol/L standard FeSO_4_ solution. The solution was shaken continuously until it turned a brownish-red color [10]. Plant N concentration was measured by Kjeldahl sulphuric acid digestion. A 0.2 g leaf sample was placed into a 100 mL dry Kettle flask. Between 0.5-1 ml of ion-free water was added to wet the sample, and 1.65 g of catalyst (K_2_SO_4_: CuSO_4_ = 10: 1; mass ratio) and 5 ml of concentrated sulphuric acid were added and the mixture was shaken gently. After digestion, the liquid was cooled and 5-10 drops of H_2_O_2_ were added slowly and shaken. Then, an Automatic Kjeldahl Analyzer was used to measure and record the volume of acid. The N concentration was calculated from the concentration of N in the test solution [11]. Plant P concentration was determined by the ammonium molybdate-antimony colorimetric method with a UV spectrophotometer [12]. A 0.25 g leaf sample was placed into a 50 mL flask, the sample was moistened with 5-10 drops of water, 8 mL of H_2_SO_4_ were added, and the mixture was shaken. Then, 10 drops of 70-72% perchloric acid (HClO_4_) were added and the mixture was shaken. After decomposition, the leaf sample was removed and cooled, and 2-3 drops of dinitrophenol indicator with NaOH (4mol/L) solution were added until the solution turned yellow. The P concentration was determined from a standard curve [10].

Leaf structural traits included leaf tissue density (LD), leaf volume (LV), leaf dry matter content (LDMC) and leaf area (LA). Leaf thickness (LT) was measured using a vernier caliper (accuracy: 0.01mm). Leaf fresh weight was measured using an electronic balance (accuracy: 0.0001g); the leaves samples were subsequently dried at 65℃ for 48 hours to determine their dry weight. LDMC was the ratio of leaf dry weight to leaf fresh weight. The leaf was scanned (Epson, V19, Nagano, Japan), and then LA was calculated by Image J software (National Institute of Health, Bethesda, ML, USA). LV was calculated by the product of leaf area and leaf thickness, and LD was calculated as leaf dry mass/leaf volume.

Leaf economic traits included leaf carbon concentration (LCC), leaf nitrogen concentration (LNC), leaf phosphorus concentration (LPC), leaf mass per area (LMA), leaf thickness (LT) and area-based photosynthetic rate (A_area_). The determination leaf element concentrations were the same as described for roots. Leaf mass per area (LMA) was calculated as leaf dry mass/leaf area. A_area_ was determined using a portable photosynthesis measurement system (LI-6400, LI-COR Biosciences, Lincoln, NE, USA). During measurements, the light intensity was set to 1500 μmol· m^-2^ · s^-1^, the air path was kept unobstructed, the CO_2_ concentration was kept at 380 ~ 390 μmol/mol, and the leaf temperature was controlled at 27℃.

**References**

1. Wright IJ, Reich PB, Westoby M, Ackerly D, Baruch Z, Bongers F, et al. The worldwide leaf economics spectrum. Nature, 2004;428(6985):821-27. <https://doi.org/10.1038/nature02403>
2. Whitman T, Aarssen LW. The leaf size/number trade-off in herbaceous angiosperms. J. Plant Ecol. 2009;3(1):49-58. <https://doi.org/10.1093/jpe/rtp018>
3. Li Y, Liu C, Sack L, Xu L, Li M, Zhang J, et al. Leaf trait network architecture shifts with species-richness and climate across forests at continental scale. Ecol. Lett. 2022;25:1442-57. <https://doi.org/10.1111/ele.14009>
4. Xiong J, Dong L, Lu J, Hu W, Gong H, Xie S, et al. Variation in plant carbon, nitrogen and phosphorus contents across the drylands of China. Funct. Ecol. 2021;36(1):174-86. <https://doi.org/10.1111/1365-2435.13937>
5. Mediavilla S, Escudero A, Heilmeier H. Internal leaf anatomy and photosynthetic resource-use efficiency: Interspecific and intraspecific comparisons. Tree Physiol. 2001;21:251-59. <https://doi.org/10.1093/treephys/21.4.251>
6. Li J, Chen X, Niklas KJ, Sun J, Wang Z, Zhong Q, et al. A whole‐plant economics spectrum including bark functional traits for 59 subtropical woody plant species. J. Ecol. 2021;110:248-61. https://doi.org/10.1371/journal.pone.0066450
7. Niinemets U. Is there a species spectrum within the world-wide leaf economics spectrum? Major variations in leaf functional traits in the Mediterranean sclerophyll *Quercus ilex*. New Phytol. 2015;205(1):79-96. https://doi.org/10.1111/nph.13001
8. Niinemets, Ü. Components of leaf dry mass per area–thickness and density–alter leaf photosynthetic capacity in reverse directions in woody plants. New Phytol. 1999;144(1):35-47. https://doi.org/10.1046/j.1469-8137.1999.00466.x
9. Niinemets, Ü. Global‐scale climatic controls of leaf dry mass per area, density, and thickness in trees and shrubs. Ecology 2001;82(2): 453-469. https://doi.org/10.1890/0012-9658(2001)082[0453:GSCCOL]2.0.CO;2
10. Black CA, Evans DD, & Dinauer R. Methods of Soil Analysis. Madison, WI: American Society of Agronomy. 1965. <https://doi.org/10.2134/agronmonogr9.1>
11. Bremner JM. Determination of nitrogen in soil by the Kjeldahl method. The Journal of Agricultural Science, 1960;55:11–33. <https://doi.org/10.1017/S0021859600021572>
12. Sparks DL, Fendorf SE, Toner CV IV, Carski TH. Kinetic Methods and Measurements. In: DL. Sparks, AL. Page, PA Helmke, RH Loeppert, PN Soltanpour, MA Tabatabai, CT Johnston and ME Sumner, editors. Methods of Soil Analysis. 1996. P. 1275-1307. https://doi.org/10.2136/sssabookser5.3.c43

**Appendix S2. The list of plant species in the study.**

| **Species** | **Genus** | **Family** |
| --- | --- | --- |
| *Anabasis aphylla* | Anabasis | Amaranthaceae |
| *Atriplex patens* | Atriplex | Amaranthaceae |
| *Atriplex sibirica* | Atriplex | Amaranthaceae |
| *Ceratoides latens* | Ceratoides | Amaranthaceae |
| *Chenopodium acuminatum* | Chenopodium | Amaranthaceae |
| *Corispermum mongolicum* | Corispermum | Amaranthaceae |
| *Corispermum puberulum* | Corispermum | Amaranthaceae |
| *Corispermum hyssopifolium* | Corispermum | Amaranthaceae |
| *Halogeton glomeratus* | Halogeton | Amaranthaceae |
| *Halostachys caspica* | Halostachys | Amaranthaceae |
| *Haloxylon ammodendron* | Haloxylon | Amaranthaceae |
| *Iljinia regelii* | Iljinia | Amaranthaceae |
| *Kali tragus* | Kali | Amaranthaceae |
| *Kalidium cuspidatum* | Kalidium | Amaranthaceae |
| *Kalidium caspicum* | Kalidium | Amaranthaceae |
| *Kalidium foliatum* | Kalidium | Amaranthaceae |
| *Salsola abrotanoides* | Salsola | Amaranthaceae |
| *Salsola ikoikovii* | Salsola | Amaranthaceae |
| *Salsola arbuscula* | Salsola | Amaranthaceae |
| *Salsola laricifolia* | Salsola | Amaranthaceae |
| *Salsola passerina* | Salsola | Amaranthaceae |
| *Salsola collina* | Salsola | Amaranthaceae |
| *Sympegma regelii* | Sympegma | Amaranthaceae |
| *Allium mongolicum* | Allium | Amaryllidaceae |
| *Cynanchum hancockianum* | Cynanchum | Apocynaceae |
| *Artemisia hedinii* | Artemisia | Asteraceae |
| *Artemisia ordosica* | Artemisia | Asteraceae |
| *Artemisia annua* | Artemisia | Asteraceae |
| *Artemisia frigida* | Artemisia | Asteraceae |
| *Artemisia xerophytica* | Artemisia | Asteraceae |
| *Artemisia desertorum* | Artemisia | Asteraceae |
| *Artemisia halodendron* | Artemisia | Asteraceae |
| *Artemisia lavandulaefolia* | Artemisia | Asteraceae |
| *Artemisia stelleriana* | Artemisia | Asteraceae |
| *Asterothamnus centraliasiaticus* | Asterothamnus | Asteraceae |
| *Carduus crispus* | Carduus | Asteraceae |
| *Cousinia affinis* | Cousinia | Asteraceae |
| *Echinops gmelini* | Echinops | Asteraceae |
| *Heteropappus altaicus* | Heteropappus | Asteraceae |
| *Stilpnolepis centiflora* | Stilpnolepis | Asteraceae |
| *Tugarinovia mongolica* | Tugarinovia | Asteraceae |
| *Lithospermum erythrorhizon* | Lithospermum | Boraginaceae |
| *Matthiola incana* | Matthiola | Brassicaceae |
| *Convolvulus gortschakovii* | Convolvulus | Convolvulaceae |
| *Ephedra sinica* | Ephedra | Ephedraceae |
| *Ephedra przewalskii* | Ephedra | Ephedraceae |
| *Euphorbia esula* | Euphorbia | Euphorbiaceae |
| *Alhagi sparsifolia* | Alhagi | Fabaceae |
| *Caragana sinica* | Caragana | Fabaceae |
| *Caragana korshinskii* | Caragana | Fabaceae |
| *Glycyrrhiza uralensis* | Glycyrrhiza | Fabaceae |
| *Lespedeza daurica* | Lespedeza | Fabaceae |
| *Medicago sativa* | Medicago | Fabaceae |
| *Oxytropis aciphylla* | Oxytropis | Fabaceae |
| *Sophora alopecuroides* | Sophora | Fabaceae |
| *Vicia amoena* | Vicia | Fabaceae |
| *Erodium stephanianum* | Erodium | Geraniaceae |
| *Lagochilus ilicifolius* | Lagochilus | Lamiaceae |
| *Nitraria tangutorum* | Nitraria | Nitrariaceae |
| *Nitraria sphaerocarpa* | Nitraria | Nitrariaceae |
| *Nitraria sibirica* | Nitraria | Nitrariaceae |
| *Peganum nigellastrum* | Peganum | Nitrariaceae |
| *Peganum harmala* | Peganum | Nitrariaceae |
| *Agropyron cristatum* | Agropyron | Poaceae |
| *Eragrostis pilosa* | Eragrostis | Poaceae |
| *Leymus chinensis* | Leymus | Poaceae |
| *Phragmites australis* | Phragmites | Poaceae |
| *Setaria viridis* | Setaria | Poaceae |
| *Stipa grandis* | Stipa | Poaceae |
| *Stipa sareptana var. krylovii* | Stipa | Poaceae |
| *Stipa capillata* | Stipa | Poaceae |
| *Tragus racemosus* | Tragus | Poaceae |
| *Calligonum mongolicum* | Calligonum | Polygonaceae |
| *Polygonum divaricatum* | Polygonum | Polygonaceae |
| *Aconitum carmichaeli* | Aconitum | Ranunculaceae |
| *Potaninia mongolica* | Potaninia | Rosaceae |
| *Reaumuria songarica* | Reaumuria | Tamaricaceae |
| *Tamarix chinensis* | Tamarix | Tamaricaceae |
| *Eremurus anisopterus* | Eremurus | Xanthorrhoeaceae |

**Table S1** List of plant traits and their categories, units and abbreviations.

| **Categories** | **Abbreviation** | **Variables** | **Units** |
| --- | --- | --- | --- |
| **Leaf economic traits** | **A_area_** | Area-based photosynthetic rate | µmol m^-2^ s^-1^ |
|  | **LMA*** | Leaf mass per area | g m^-2^ |
|  | **LT*** | Leaf thickness | Mm |
|  | **LCC*** | Leaf carbon concentration | g kg^-1^ |
|  | **LNC*** | Leaf nitrogen concentration | g kg^-1^ |
|  | **LPC*** | Leaf phosphorus concentration | g kg^-1^ |
| **Plant chemical traits** | **RCC** | Root carbon concentration | g kg^-1^ |
|  | **RNC** | Root nitrogen concentration | g kg^-1^ |
|  | **RPC** | Root phosphorus concentration | g kg^-1^ |
|  | **SCC** | Stem carbon concentration | g kg^-1^ |
|  | **SNC** | Stem nitrogen concentration | g kg^-1^ |
|  | **SPC** | Stem phosphorus concentration | g kg^-1^ |
| **Leaf structural traits** | **LD*** | Leaf tissue density | g cm^-3^ |
|  | **LV** | Leaf volume | cm^3^ |
|  | **LDMC*** | Leaf dry matter content |  |
|  | **LA** | Leaf area | cm^2^ |

* belongs to multiple categories. Leaf thickness (LT), Leaf mass per area (LMA), leaf tissue density (LD) and leaf dry matter content (LDMC) are in categories of both leaf economic traits and structural traits. Leaf carbon concentration (LCC), leaf nitrogen concentration (LNC) and leaf phosphorus concentration (LPC) are in categories of both leaf economic traits and chemical traits.

**Table S2** Variation in the degree of plant trait networks (PTNs) for all species among different plant traits.

| **Trait** | **Mean** | **Standard deviation** | **Standard error** | **Minimum** | **Maximum** |
| --- | --- | --- | --- | --- | --- |
| **RCC** | 4.79 | 1.595 | 0.0226 | 0 | 10 |
| **RNC** | 4.26 | 1.177 | 0.0167 | 1 | 9 |
| **RPC** | 4.38 | 1.536 | 0.0217 | 1 | 10 |
| **SCC** | 4.45 | 1.509 | 0.0213 | 1 | 10 |
| **SNC** | 8.17 | 1.740 | 0.0246 | 2 | 13 |
| **SPC** | 8.83 | 1.021 | 0.0144 | 5 | 13 |
| **LCC** | 5.18 | 1.570 | 0.0222 | 1 | 10 |
| **LNC** | 6.47 | 1.093 | 0.0155 | 2 | 10 |
| **LPC** | 7.03 | 1.146 | 0.0162 | 4 | 11 |
| **A_area_** | 2.10 | 1.401 | 0.0198 | 0 | 8 |
| **LMA** | 7.69 | 1.370 | 0.0194 | 4 | 13 |
| **LT** | 8.24 | 1.469 | 0.0208 | 4 | 13 |
| **LA** | 6.64 | 1.338 | 0.0189 | 2 | 12 |
| **LDMC** | 2.40 | 0.934 | 0.0132 | 0 | 5 |
| **LV** | 6.56 | 1.198 | 0.0169 | 3 | 11 |
| **LD** | 5.74 | 1.191 | 0.0169 | 3 | 10 |

**Table S3** Comparison of the relative importance of structural, economic and chemical traits for all species, different plant life-forms and arid regions.

| **Group** | **Structural traits** | **Economic traits** | **Chemical traits** |
| --- | --- | --- | --- |
| **PTNs-all species** | 0.36 ± 0.0006 a | 0.41 ± 0.0007 b | 0.39 ± 0.0007 c |
| **PTNs-woody plants** | 0.32 ± 0.0006 a | 0.34 ± 0.0007 b | 0.33 ±0.0006 c |
| **PTNs-herbaceous plants** | 0.47 ± 0.0005 a | 0.43 ± 0.0005 b | 0.40 ± 0.0006 c |
| **PTNs-arid regions** | 0.30 ± 0.0004 a | 0.34 ± 0.0005 b | 0.31 ± 0.0005 c |
| **PTNs-semi-arid regions** | 0.45 ± 0.0008 a | 0.54 ± 0.0008 b | 0.52 ± 0.0008 c |

Means with different lower-case within a row differ from each other (*P* < 0.05). PTNs-all species: plant trait networks of all plants; PTNs-woody plants: plant trait networks of woody plants; PTNs-herbaceous plants: plant trait networks of herbaceous plants; PTNs-arid regions: plant traits networks of arid regions; PTNs-semi-arid regions: plant traits networks of semi-arid regions.

**Table S4** Variation in the degree of plant trait networks (PTNs) for all species among different plant life-forms.

| **Group** | **Trait** | **Mean** | **Standard deviation** | **Standard error** | **Minimum** | **Maximum** |
| --- | --- | --- | --- | --- | --- | --- |
| **PTNs-woody plants** | **RCC** | 4.69 | 1.205 | 0.0170 | 1 | 9 |
|  | **RNC** | 4.47 | 0.862 | 0.0122 | 1 | 8 |
|  | **RPC** | 3.25 | 1.136 | 0.0161 | 1 | 8 |
|  | **SCC** | 2.57 | 1.050 | 0.0149 | 1 | 7 |
|  | **SNC** | 6.65 | 1.527 | 0.0216 | 3 | 12 |
|  | **SPC** | 7.89 | 1.102 | 0.0156 | 3 | 11 |
|  | **LCC** | 3.40 | 1.355 | 0.0192 | 1 | 9 |
|  | **LNC** | 4.80 | 1.421 | 0.0201 | 2 | 10 |
|  | **LPC** | 5.13 | 1.228 | 0.0174 | 2 | 9 |
|  | **A_area_** | 3.81 | 1.383 | 0.0196 | 0 | 9 |
|  | **LMA** | 6.63 | 0.948 | 0.0134 | 4 | 10 |
|  | **LT** | 6.92 | 1.362 | 0.0193 | 3 | 10 |
|  | **LA** | 5.19 | 0.599 | 0.0085 | 3 | 8 |
|  | **LDMC** | 2.15 | 1.022 | 0.0145 | 0 | 6 |
|  | **LV** | 5.38 | 1.499 | 0.0212 | 3 | 11 |
|  | **LD** | 6.20 | 1.113 | 0.0158 | 4 | 10 |
| **PTNs-herbaceous plants** | **RCC** | 6.13 | 1.619 | 0.0229 | 0 | 10 |
|  | **RNC** | 4.07 | 1.164 | 0.0165 | 0 | 10 |
|  | **RPC** | 4.58 | 0.991 | 0.0140 | 3 | 10 |
|  | **SCC** | 6.38 | 1.556 | 0.0220 | 2 | 12 |
|  | **SNC** | 7.87 | 1.367 | 0.0193 | 3 | 12 |
|  | **SPC** | 7.04 | 1.661 | 0.0235 | 2 | 11 |
|  | **LCC** | 6.03 | 1.544 | 0.0218 | 2 | 11 |
|  | **LNC** | 8.11 | 1.114 | 0.0158 | 5 | 12 |
|  | **LPC** | 6.98 | 0.886 | 0.0125 | 4 | 11 |
|  | **A_area_** | 4.58 | 0.989 | 0.0140 | 1 | 7 |
|  | **LMA** | 7.12 | 1.513 | 0.0214 | 2 | 11 |
|  | **LT** | 5.99 | 0.673 | 0.0095 | 3 | 10 |
|  | **LA** | 6.97 | 1.045 | 0.0148 | 3 | 11 |
|  | **LDMC** | 3.16 | 0.844 | 0.0119 | 0 | 6 |
|  | **LV** | 8.96 | 0.938 | 0.0133 | 5 | 12 |
|  | **LD** | 8.82 | 1.252 | 0.0177 | 5 | 12 |

PTNs-woody plants: plant trait networks of woody plants; PTNs-herbaceous plants: plant trait networks of herbaceous plants.

**Table S5** Variation in the degree of plant trait networks (PTNs) for all species among different arid regions.

| **Group** | **Trait** | **Mean** | **Standard deviation** | **Standard error** | **Minimum** | **Maximum** |
| --- | --- | --- | --- | --- | --- | --- |
| **PTNs-arid regions** | **RCC** | 2.79 | 1.201 | 0.0170 | 0 | 7 |
|  | **RNC** | 3.69 | 0.761 | 0.0108 | 1 | 7 |
|  | **RPC** | 2.77 | 1.093 | 0.0155 | 1 | 7 |
|  | **SCC** | 3.24 | 1.014 | 0.0143 | 0 | 8 |
|  | **SNC** | 7.13 | 1.553 | 0.0220 | 2 | 12 |
|  | **SPC** | 8.06 | 0.850 | 0.0120 | 5 | 12 |
|  | **LCC** | 4.00 | 1.540 | 0.0218 | 0 | 9 |
|  | **LNC** | 6.48 | 0.813 | 0.0115 | 3 | 11 |
|  | **LPC** | 5.96 | 1.056 | 0.0149 | 4 | 10 |
|  | **A_area_** | 0.97 | 1.029 | 0.0146 | 0 | 6 |
|  | **LMA** | 6.47 | 1.065 | 0.0151 | 4 | 10 |
|  | **LT** | 7.16 | 1.104 | 0.0156 | 5 | 12 |
|  | **LA** | 5.18 | 0.981 | 0.0139 | 2 | 9 |
|  | **LDMC** | 2.13 | 0.830 | 0.0117 | 0 | 6 |
|  | **LV** | 5.71 | 0.852 | 0.0121 | 3 | 9 |
|  | **LD** | 5.07 | 0.799 | 0.0113 | 3 | 8 |
| **PTNs-semi- arid regions** | **RCC** | 7.45 | 1.365 | 0.0193 | 4 | 14 |
|  | **RNC** | 8.86 | 1.049 | 0.0148 | 5 | 14 |
|  | **RPC** | 7.88 | 1.297 | 0.0183 | 3 | 14 |
|  | **SCC** | 8.09 | 1.693 | 0.0239 | 3 | 14 |
|  | **SNC** | 7.52 | 1.542 | 0.0218 | 4 | 13 |
|  | **SPC** | 6.76 | 1.557 | 0.0220 | 4 | 14 |
|  | **LCC** | 9.92 | 1.542 | 0.0218 | 3 | 15 |
|  | **LNC** | 9.39 | 1.562 | 0.0221 | 3 | 13 |
|  | **LPC** | 7.72 | 1.408 | 0.0199 | 3 | 14 |
|  | **A_area_** | 5.81 | 1.576 | 0.0223 | 1 | 13 |
|  | **LMA** | 7.42 | 1.730 | 0.0245 | 1 | 13 |
|  | **LT** | 8.48 | 1.684 | 0.0238 | 3 | 13 |
|  | **LA** | 8.39 | 1.193 | 0.0169 | 5 | 13 |
|  | **LDMC** | 3.79 | 0.901 | 0.0127 | 1 | 9 |
|  | **LV** | 9.47 | 1.301 | 0.0184 | 6 | 14 |
|  | **LD** | 5.06 | 1.709 | 0.0242 | 2 | 12 |

PTNs-arid regions: plant traits networks of arid regions; PTNs-semi-arid regions: plant traits networks of semi-arid regions.

**Figure. S1** The sampling sites (black dots) in the drylands of China.


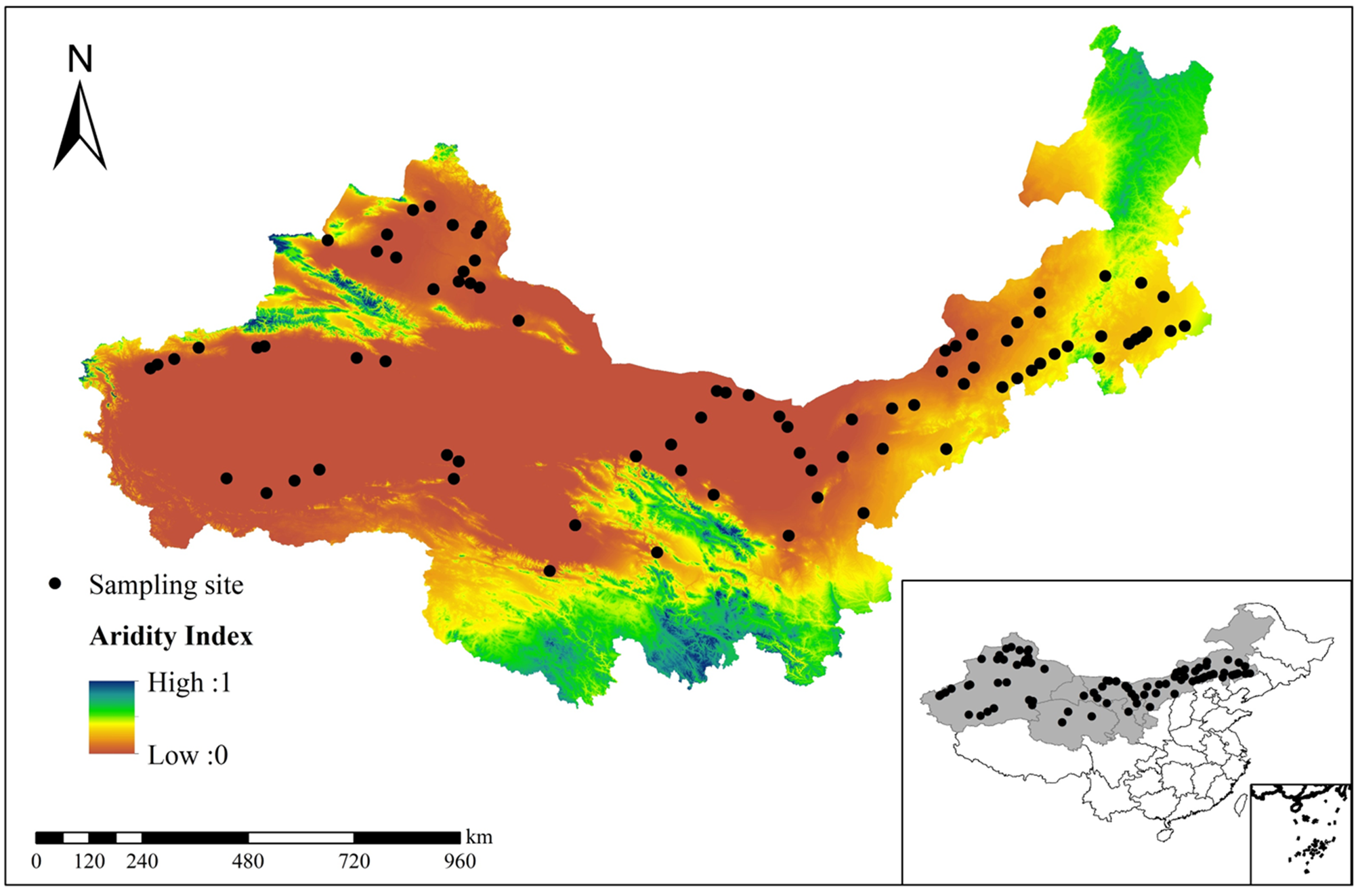


**Figure. S2** Relationships of plant trait network-level parameters to the number of species for simulated networks. For a given number of species, we randomly sampled species from the species pool (188 species), and 500 PTNs were determined and their PTN-level parameters were calculated. Each point is the mean of 500 values. The red lines were fitted using linear regressions.


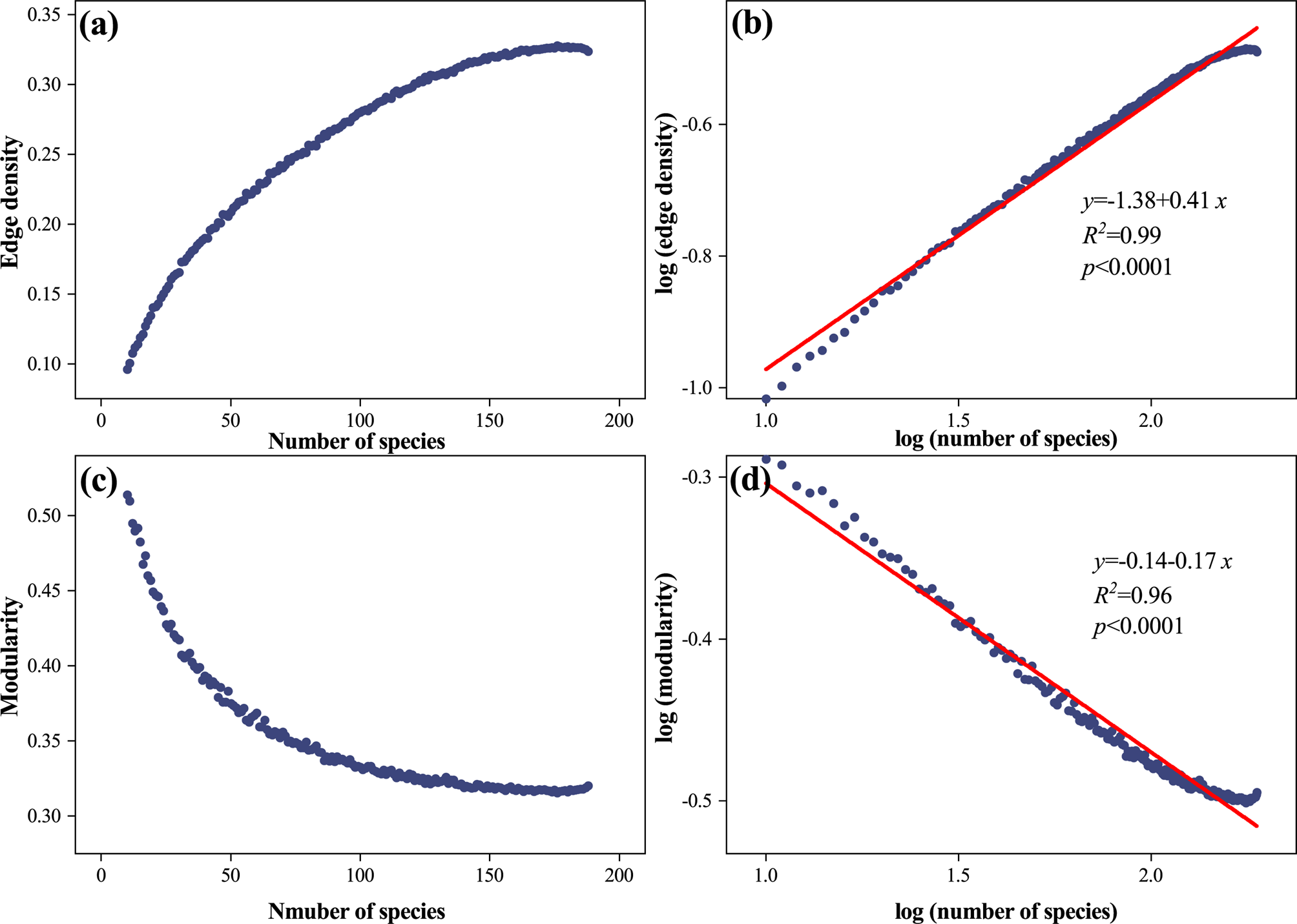


**Figure. S3** Differences in the modularity (a) and edge density (b) of plant trait networks (PTNs) between different plant life-forms. Means with different letters differ from each other (*P* < 0.05). Error bars represent standard error (SE).


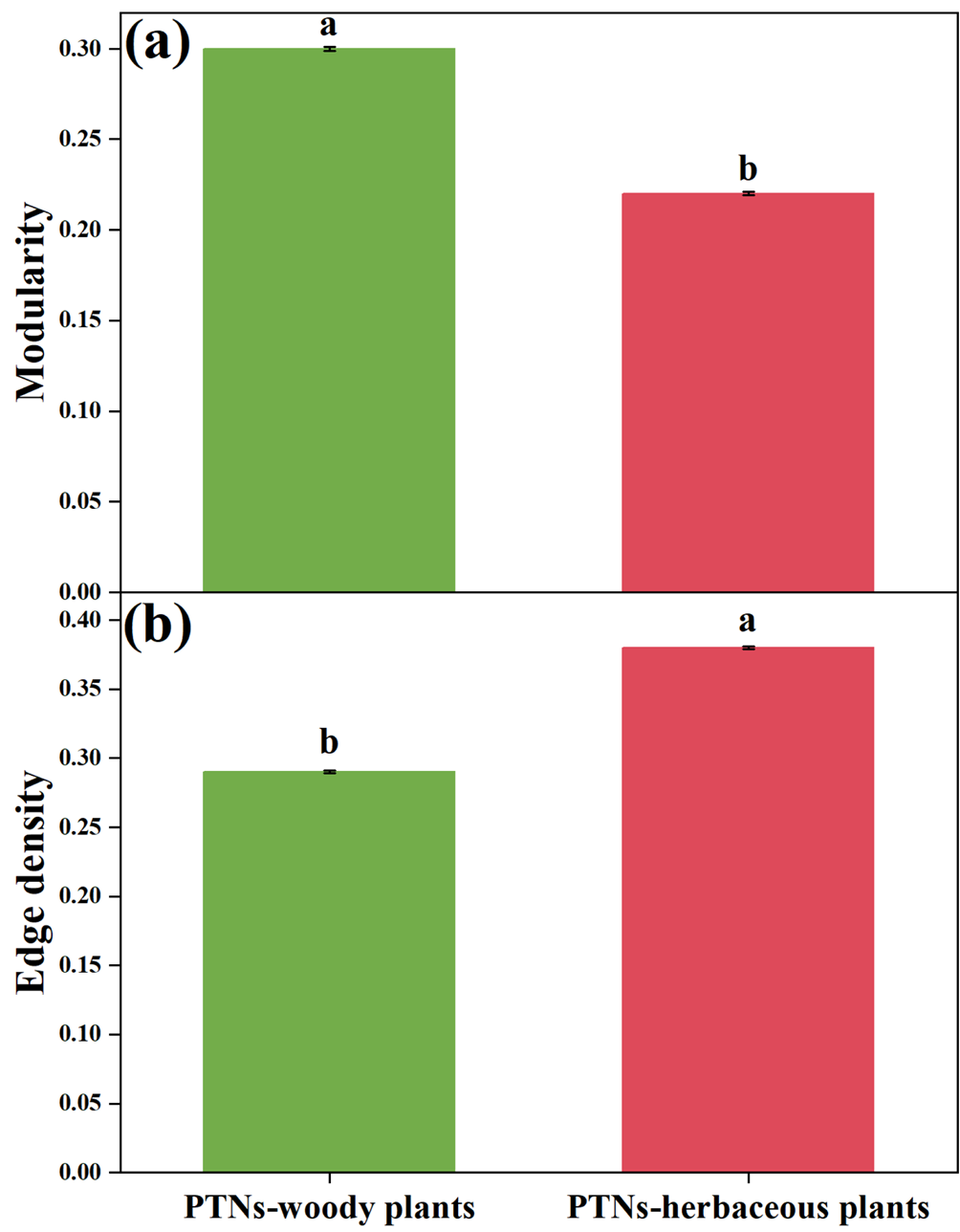


**Figure. S4** Differences in the modularity (a) and edge density (b) of plant trait networks (PTNs) between different arid regions. Means with different letters differ from each other (*P* < 0.05). Error bars represent standard error (SE).


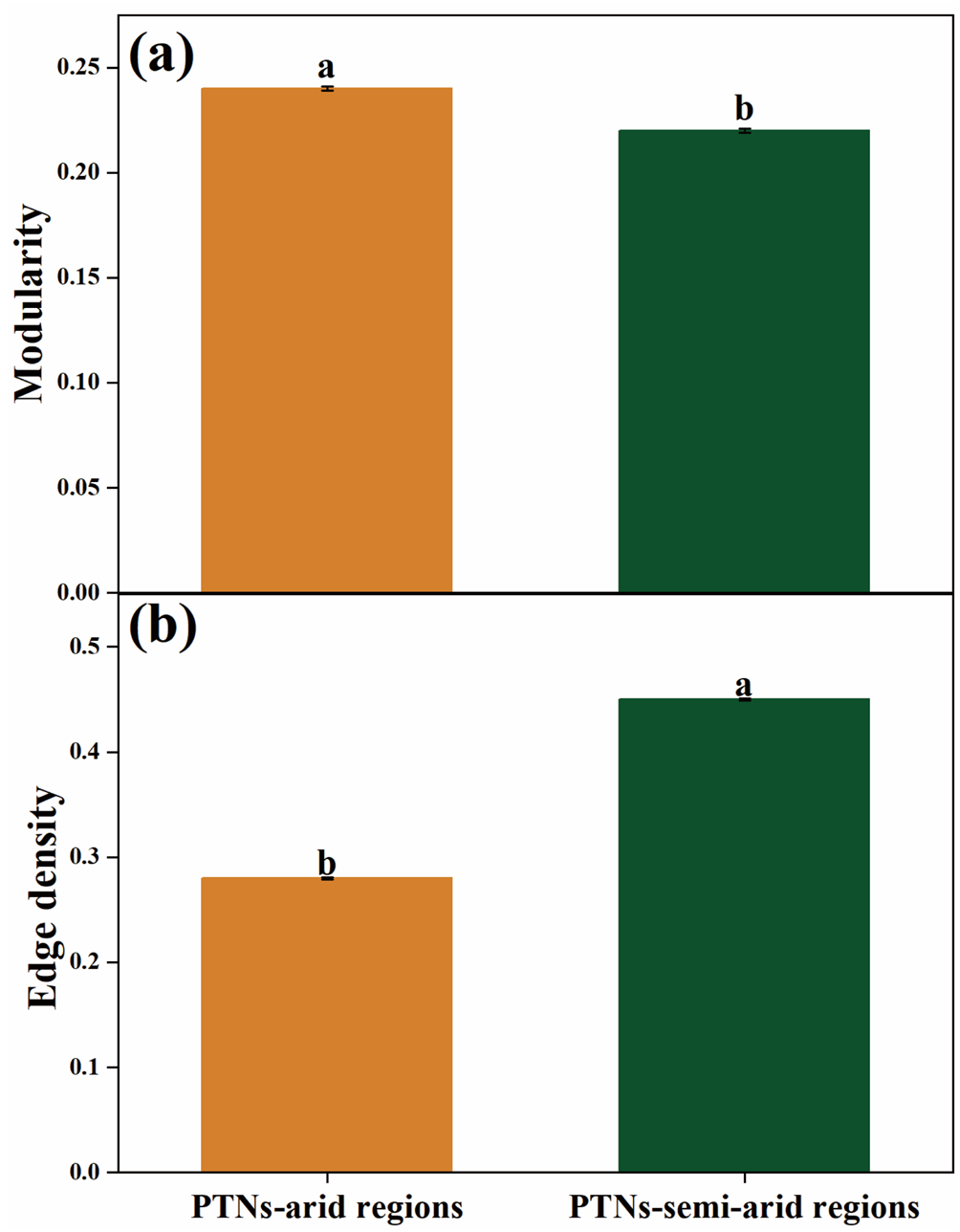

Supplement: Supplementary file 1 — Supplementary Material 1 [file 12870_2023_4273_MOESM1_ESM.docx]
